# Minimalistic Approach to Coreference Resolution in Lithuanian Medical Records

**DOI:** 10.1155/2019/9079840

**Published:** 2019-03-20

**Authors:** Voldemaras Žitkus, Rita Butkienė, Rimantas Butleris, Rytis Maskeliūnas, Robertas Damaševičius, Marcin Woźniak

**Affiliations:** ^1^Faculty of Informatics, Kaunas University of Technology, 51386 Kaunas, Lithuania; ^2^Institute of Mathematics, Silesian University of Technology, 44-100 Gliwice, Poland

## Abstract

Coreference resolution is a challenging part of natural language processing (NLP) with applications in machine translation, semantic search and other information retrieval, and decision support systems. Coreference resolution requires linguistic preprocessing and rich language resources for automatically identifying and resolving such expressions. Many rarer and under-resourced languages (such as Lithuanian) lack the required language resources and tools. We present a method for coreference resolution in Lithuanian language and its application for processing e-health records from a hospital reception. Our novelty is the ability to process coreferences with minimal linguistic resources, which is important in linguistic applications for rare and endangered languages. The experimental results show that coreference resolution is applicable to the development of NLP-powered online healthcare services in Lithuania.

## 1. Introduction

Digital means of medical informatics, especially when applying natural language processing (NLP), are indispensable in the application of e-health and digitalization of medical records and processes [1]. The use of NLP has proved as a lower-cost alternative to traditional medical methods in many cases such as to forecast stress symptoms and suicide risk in free-text responses sent via a mobile phone [2], or to detect seasonal disease outbreaks by monitoring search engine queries [3], and discovery of healthcare knowledge from social media [4, 5].

With the development of Semantic Web technology, web information retrieval (IR) is changing towards meaning-based IR. The quality of retrieved documents relevant to the user also highly depends on the information extraction (IE) methods applied. In general, IE focuses on automatic extraction of structured information from the unstructured source. Standard document text preprocessing steps used in IE are lexical analysis, morphological analysis, and named entity recognition (NER), which can be complemented by coreference resolution and semantic annotation. The main issue here is the ambiguity and complexity of the natural language, thus making the progress in IE dependent on the evolution of the NLP techniques. While for widely used languages (such as English), the IE-related NLP research has already reached the levels of maturity and practical application on a massive scale (e.g., IBM Watson project) [6], but the resource-poor languages, such as Lithuanian [7], remain an open NLP research field. The baseline application is often steered towards automated concept extraction [8, 9], often in combination with text mining [10, 11].

NER when applied to biomedical texts is a critical step for developing decision support tools for smart healthcare. Examples for it are as follows: drug name recognition (DNR), which recognizes pharmacological substances from biomedical texts and classifies them for discovering drug-drug interactions [12, 13]; biomedical named entity recognition (BNER), which extracts biomedical concepts of interest such as genes and proteins [14]; and medical entity recognition, which is information extraction from unstructured electronic health records [15–17]. Such studies include mapping clinical descriptions to Systematized Nomenclature of Medicine codes [18] or other medical lexicons. Unstructured texts in the medical domain contain valuable medical information, and there are many errors, such as spelling errors, improper grammatical use, and semantic ambiguities, which hinder data processing and analysis [19]. Structuration of medical domain knowledge using biomedical ontologies and controlled vocabularies provide support for data standardization and interoperability, healthcare administration, and clinical decision support [20]. Rich concepts linked by semantic relationships such as in the Unified Medical Language System (UMLS) contribute to healthcare data integration, pattern mining from EHRs, medical entity recognition in clinical text, and clinical data sharing [21]. The development of online healthcare services as powerful platforms provides users with an opportunity to address health concerns such as improving patient-centered care and supporting self-management. The users consider online healthcare services as a vital source of health information but still need more powerful semantic search engines to arrive at informed decisions on their own health and for more active participation in healthcare processes [22]. However, these services depend greatly upon the support provided by the natural language processing.

Our previous work included the development of the semantic search framework [23] for answering questions presented in structured Lithuanian language, which is based on Semantics of Business Vocabulary and Business Rules (SBVR) language. Our results in [23] showed that there is a strong need to complement the NLP pipeline of semantic search with the coreference resolution. Such coreference resolution tools have not been developed for the Lithuanian language yet, especially for very sector-important digital medical application, as we apply our algorithm to process digital transcripts of a hospital reception. Therefore, the creation of such tools is a prerequisite for further improvement of NLP-supported health-oriented decision making in the Lithuanian language, while the experience gained could be extended to developing semantic search tools for other under-resourced languages as well.

The rest of the paper is structured as follows. In Section 2, we analyse the related works in the coreference resolution field, including the state-of-the-art and the methods proposed for languages which are grammatically similar to Lithuanian. Sections 3 and 4 present the coreference resolution algorithm and its experimental evaluation.  Section 5 presents conclusions and discusses future work.

## 2. Related Works

Machine-learning and rule-based approaches are efficient methods in semantic processing, especially when enhanced with external knowledge and coreference clues derived from the structured document, while often still performing better (in comparison with classic implementations) in coreference resolution when provided with ground truth mentions [24], while further expanded with scaffolding approaches [25]. Unsupervised methods can be applied to large-scale scenarios [26, 27]. Alternatively, a hybrid strategy may be used based on a set of statistical measures and syntactical and semantic information [28]. “Off the shelf” type of IR algorithms can be utilized quite successfully in some of the scenarios, especially with limited focus areas (in a medical sense) [29]. Accuracy can be further improved by analysing trigram frequencies [30] and applying graph-style algorithms [31] in context-sensitive corpus fragments.

In general, the coreference resolution methods can be classified into knowledge rich and knowledge poor. Both methods require large resources such as semantic information, syntactic annotations, or preannotated corpora of hospital transcripts from a hospital reception. Under resourced, rarer languages, like Lithuanian, usually do not have such resources available.

While up-to-date, no research has been performed to solve coreferences in Lithuanian, but many solutions have been proposed for other languages, mostly for English (Table 1). Note that the evaluation results are not directly comparable, as the authors used different corpora.

Considering languages that are more-or-less grammatically similar to Lithuanian (which is one of the Baltic languages), we summarize the related work on Latvian (only other Baltic language) and Slavic languages such as Polish, Russian, and Czech in Table 2.

- For Latvian, the only solution is LVCoref [45]. It is a rule-based system that uses an entity-centric model. It focuses on named entity matches (exact matches, acronyms) and uses Hobbs' algorithm for pronouns.
- For Polish, rule-based Ruler [46] for scoring of candidates uses coreferences gender/number and including (removal of nested groups) rules, lemma, and Wordnet rules for nominal expressions and pronoun rule specifically targeting pronouns. BARTEK [47] is an adaptation of BART, which was designed for English, to Polish. Mixed Polish coreferences resolution approach combines neural networks architecture with the sieve-based approach [48].
- For Russian, RU-EVAL-2014 [49] was an evaluation campaign of anaphora and coreferences resolution tools that employed a wide variety of approaches. The evaluation was performed on Russian Coreference Corpus (RuCur). Machine learning approaches [50] were also used.
- For Czech, coreferences are annotated in the tectogrammatical layer of Prague Dependency Treebank (PDT) and their first coreference resolution approach was rule based [51]. At first, all possible candidates are collected and then their list is narrowed down using 8 filters, and then from remaining ones closest to corefering object is selected as antecedent. Nguy et al. [52] adapted two older English language approaches to Czech language and used Decision Tree C5 for the classifier-based approach, while the ranker-based approach employed the averaged perceptron algorithm. Both approaches were trained and evaluated on PTD data with ranker-based approach providing better results. Treex CR [53] was developed for the Czech language and adapted to English, Russian, and German, although for Russian and German, English coreferences labels were projected, which produced notably lower results [54].

In summary, the rule-based solutions have the advantages of easier adaptability and provide comparable results when good training data are not available as is the case for Lithuania. Many of more advanced solutions cannot be fully adapted for rarer and under-resourced languages due to the lack of available linguistic resources, as is the case with Lithuanian language. For example, BART at the time supported 64 feature extractors, but due to lack of language-specific resources for the Polish language, only 13 could be utilized. The solutions that are not heavy on linguistic resources can be very useful for resource-poor languages in general.

## 3. A Rule-Based Coreference Resolution: A Lithuanian Case

### 3.1. Definition and Framework

Coreference resolution (or anaphora) is an expression, the interpretation of which depends on another word or phrase presented earlier in the text (antecedent). For example, “Tom has a backache. He was injured.” Here the words “Tom” and “He” refer to the same entity. Without resolving the relationship between these two structures, it would not be possible to determine why Tom has the backache, nor who was injured. In such cases, semantic information would be lost.

Anaphoric objects are expressed with pronouns and cannot be independently interpreted without going back to its antecedent. In this work, such expressions are called coreferences, unless it is required to make a distinction. Usage of such expressions can vary depending on the type and the style of the text. Here we focus on texts from medical-related domains.

**Figure 1:**
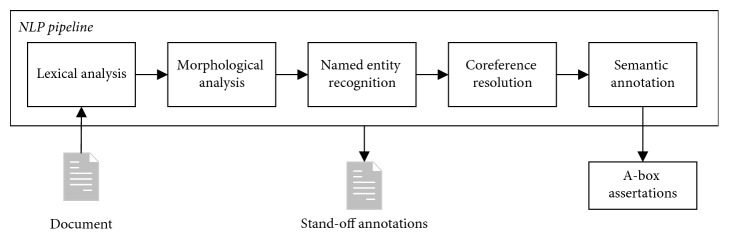
An NLP pipeline of semantic search framework with coreference resolution component.

The role of coreference resolution in the semantic search framework is to provide additional semantic information after named entity recognition before semantic annotation (Figure 1).

### 3.2. Conceptual Model of Coreference Resolution

**Figure 2:**
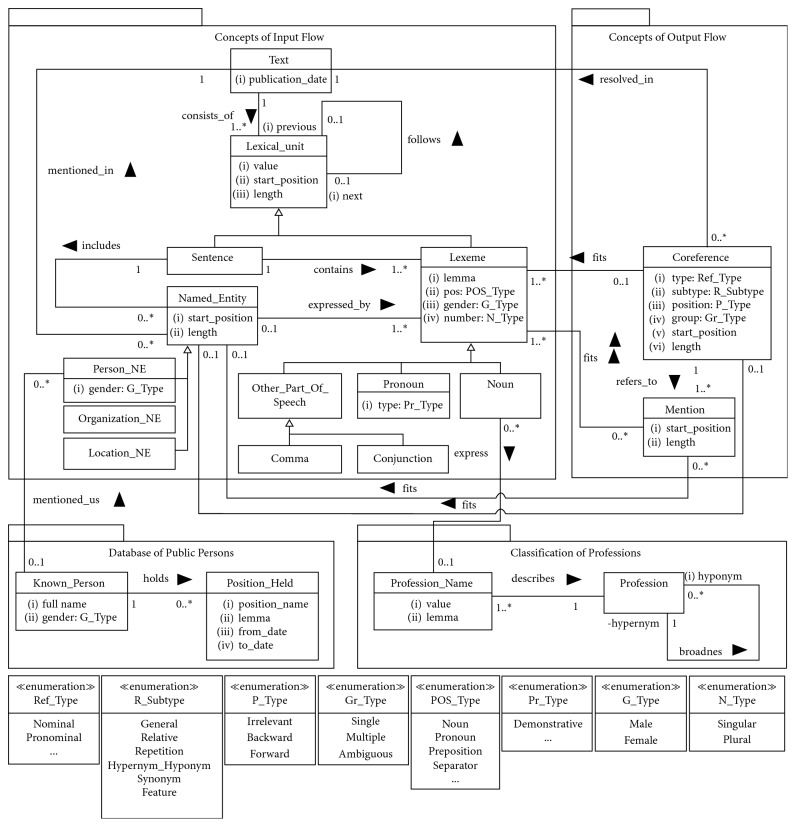
A conceptual model of a coreference resolution domain.

In this chapter, a conceptualization of coreference resolution is presented. A given model, which is expressed as UML class diagram (Figure 2), specifies the concepts playing a certain role in the extraction of coreferences of a certain type. The model gives us an understanding of the following:

- What features of text, sentence, and word help us recognize the existence of coreference (they are specified in the package Concepts of Input Flow)
- What kind of text preprocessing is required
- What additional resources are required for resolution of certain type coreferences (they are specified in the package Database of Public Persons and Classification of Professions)

For example, from the model provided, it is clear that, before coreference resolution starts, it is important to preprocess text and obtain the following:

- A text segmented into sentences and lexemes
- Morphological features of lexemes identified
- Named entities recognized

Text preprocessing itself is not a task of coreference resolution, so it is out of the scope of this paper.

It is worthy to mention that the model is quite abstract, language independent, and technology independent. Therefore, it is applicable not only for Lithuanian but for grammatically similar languages as well. Concepts of this model are used for the formalization of coreference resolution rules in the next section. The concepts are explained in more detail below.

The main concepts of coreference resolution are *Text*, *Lexical_Unit*, and *Named_Entity*. The concept *Text* assumes a textual document whose content should be analysed. Each test has an associated publication date, which is important for solving coreferences. Each text consists of at least one *Lexical_Unit*, which includes paragraphs, sentences, words, and punctuations, classified into the *Sentence* and *Lexeme* categories. *Lexeme* assumes lexical units such as words, punctuations, and numbers. Each lexeme is characterized by a lemma and a part of speech, and some of them (nouns and pronouns) by grammatical gender and number. The lexeme could be specialized by POS category: *Noun*, *Pronoun,* and *Other_Part_Of_Speech*. Special cases of *Other_Part_Of_Speech* are *Comma* and *Conjunction,* which are required for the description of conditions of some coreference resolution rules.

A *Named_Entity* concept defines an object to whom pronouns or certain nouns can refer. NER algorithms usually recognize three types of entities: a person (*Person_NE*), an organization (*Organization_NE*), and a location (*Location_NE*). The named entities of a person type require special attention a person can be mentioned not only using pronouns but also using a position he/she holds (*Position_Held*) and a professional name (*Profession)*. Additional information about a person could help resolving such coreferences more precisely. As an example, source of such information could be a Database of Public Person, which includes *Known_Person*—a well-known person mentioned as *Person_NE* in the text. The output of a coreference resolution algorithm is a *Coreference*—a relationship between coreferents. For each coreference, its type (nominal and pronominal), subtype (relative pronoun and noun repetition), position (points backward, forward, or irrelevant in case of repetitions), and group (is singular, refers to the coreference group or is ambiguous) are specified. Each referent refers at least to one coreferent (a concept *Mention*). Each *Mention* starts at a certain position in the text, is of a certain length, and fits at least one *Lexeme*. Some of them can fit a certain *Named_Entity*.

### 3.3. Coreference Resolution Algorithms

**Figure 3:**
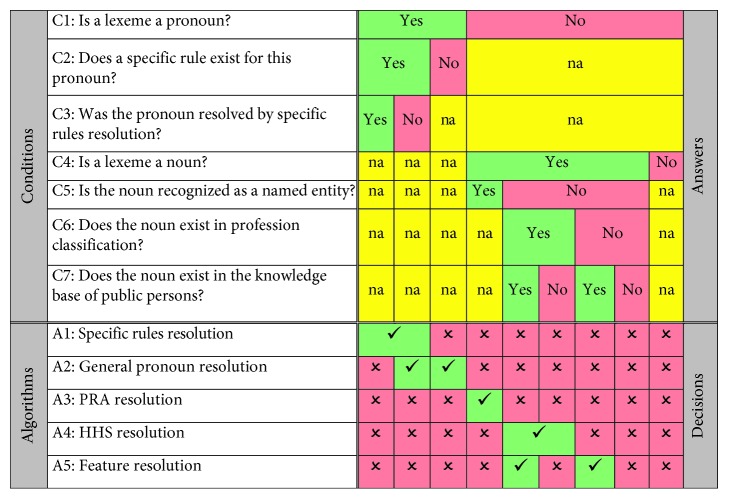
A decision table for selection of the algorithm. na: not applicable; 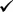: the algorithm should be applied; 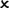: it should not be applied.

The decision table with guidelines for the application of the certain resolution algorithm is shown as Figure 3. The conditions are checked consecutively on every lexeme in the text, and, if the condition is satisfied, a corresponding algorithm is activated. For example, if C2 condition is met then immediately A1 algorithm is activated.

For resolution of a specific type of references, we propose the following algorithms:

- A1: specific rules resolution algorithm for resolution of certain usage of pronouns
- A2: general pronoun resolution algorithm which focuses on the cases where pronouns refer to nouns (or noun phrases) that are recognized as named entities of “person” class
- A3: PRA (partial, repetition, and acronym) resolution algorithm for resolution of nouns recognized as named entities and their repeated usage in the same text
- A4: HHS (hypernym, hyponym, synonymous) resolution algorithm for resolution of nouns recognized as profession names including their synonyms and hypernyms/hyponyms
- A5: feature resolution algorithm for resolution of nouns that represent certain feature (at the moment only public position being held) of the named entity of a person

The coreference resolution starts from the sequential analysis of each lexeme looking for a certain type of pronoun and noun. Depending on identified features of lexeme, a decision about further analysis is taken. The decision table (Figure 3) summarizes conditions for the application of the certain resolution algorithm. The conditions are listed in the upper left quadrant; the decision alternatives are listed in the lower left quadrant. The upper right quadrant shows the possible alternatives for the conditions of the corresponding row. In the upper right quadrant, the answer “na” stands for “not relevant.” In the lower right quadrant, “✓” means that the algorithm should be applied and “✗” means that it should not be applied.

The idea is that the pronoun-related coreferences should be solved first sequentially by checking the conditions C1, C2, and C3. Then a noun-related coreference resolution should start by sequentially checking the conditions C4, C6, and C7.

### 3.4. Formal Description of Coreference Resolution Algorithms

First-order logic (FOL) formulas are employed to define the main conditions the algorithms should check when resolving coreferences. The concepts of the coreference resolution model (Figure 2) became the predicates or constants in the FOL formulas: the classes became the unary predicates of the same name as class; the associations between classes—the binary predicates of the same name as association; the attributes of classes—the binary predicates of the same name as attribute plus verb “has” at the beginning; and the literals of enumerations—constants.

The algorithms follow the grammar rules of the Lithuanian language which are based on the analysis of morphological features of lexemes and their order in the sentence and text. Examples of Lithuanian language sentences were translated into English as closely as possible. All proper names were changed to generic abbreviations to comply with GDPR.

#### 3.4.1. A1: Specific Rules Resolution

In some cases, there exists a rather rigid structure for pronoun usage and it can be easily defined by using specific rules, for example,

- [LT] Šiandien buvo atėjęs **vyras** [noun], **kuris** [pronoun] skundėsi nugaros skausmu.
- [EN] A **man** [noun] **who** [pronoun] had a backache came today.
- [LT] Šiandien buvo atėjęs **vyras** [noun], **su** [preposition] **kuriuo** [pronoun] aptarėme nugaros skausmą.
- [EN] A **man** [noun] **with** [preposition] **whom** [pronoun] we discussed a backache came today.

Both examples are similar in their construction: [noun] [comma] [optional preposition] [specific pronoun]. In both cases, pronoun “kuriuo” refers to the noun “vyras.” In the first example, we do not have an optional preposition “su,” while we have it in the second one.

A condition for the existence of such reference formally is defined as follows:

*For every sentence* **s** *of text* **t** *and for every “Relative” type pronoun* **p** *, which is contained in the sentence* **s** *and has a start position* **sp1** *, is of length* **ln1** *, follows comma* **c** *or follows prepositional lexeme* **l1** *, which follows comma* **c** *, and for every noun* **l2** *, which has a start position* **sp2** *, is of length* **ln2** *, precedes comma* **c** *, is of the same gender* **g** *and of the same number* **n** *as the pronoun* **p** *, the only one coreference relation* **r** *, which is resolved in text* **t** *, is of “Pronominal” type, “Relative” subtype, “Backward” position and “Single” group between the pronoun* **p** *and the noun* **n** *, its referent starts at position* **sp1** *and has length* **ln1** *, and which fits only one lexeme* **p** *and refers to only one mention* **m** *, which starts at position* **sp2** *, has length* **ln2** *, and fits only one lexeme* **l2** *, exists* (Rule 1).

  Rule 1: ∀t, s, p, l1, c, l2, g, n, sp1, sp2, ln2. [Text(t) ⋀ Sentence(s) ⋀ consists_of(t, s) ⋀ Pronoun(p) ⋀ contains(s, p) ⋀ has_type(p, Relative) ⋀ has_start_position(p, sp1) ⋀ has_length(p, ln1) ⋀ Comma(c) ⋀ (follows(p, c) ⋁ (Lexeme(l1) ⋀ has_pos(l1, Preposition) ⋀ follows(l1, c) ⋀ follows(p, l1)) ⋀ Noun(l2) ⋀ follows(l2, c) ⋀ has_gender (p, g) ⋀ has_gender (l2, g) ⋀ has_number(p, n) ⋀ has_number(l2, n) ⋀ has_start_position(l2, sp2) ⋀ has_length(l2, ln2) ⟶ ∃!r ∃!m. [Coreference(r) ⋀ resolved_in(r, t) ⋀ has_type(r, Pronominal) ⋀ has_subtype(r, Relative) ⋀ has_position(r, Backward) ⋀ has_ group(r, Single) ⋀ has_start_position(r, sp1) ⋀ has_length(r, ln1) ⋀ fits(r, p) ⋀ Mention(m) ⋀ refers_to(r, m) ⋀ has_start_position(m, sp2) ⋀ has_length(m, ln2) ⋀ fits(m, l2)]]

The relative pronoun might be plural and refer to multiple singular (or multiple plural) nouns:

- [LT] Komisija nerado panašumų tarp **Tomo** [noun], **Lino** [noun], **Petro** [noun] **ir** [conjunction] **Eglės** [noun], **kurių** [pronoun] sužalojimai atrodė panašūs.
- [EN] The committee did not find **Tom** [noun], **Linas** [noun], **Peter** [noun] **and** [conjunction] **Eglė** [noun], **who** [pronoun] shared similar injuries.

In this case, a plural pronoun “kurių” is referring to four singular nouns that have different genders. The previous rule would not be able to solve such coreference. For this case, the construction would be: [noun] [comma] [noun] [comma] [noun] [conjunction] [noun] [comma] [optional preposition] [specific pronoun].

For such case, a special condition must be defined:

*For every sentence* **s** *in text* **t** *and for every “Relative” type pronoun* **p** *of “Plural” number, which is contained in the sentence* **s** *and has a start position* **sp1** *, is of length* **ln1** *, follows comma* **c1** *or follows prepositional lexeme* **l** *, which follows comma* **c1** *, and for every noun* **n1** *, which precedes comma* **c1** *, has a start position* **sp2** *, is of length* **ln2** *, follows conjunction* **j** *, and for every noun* **n2** *, which precedes conjunction* **j** *, has a start position* **sp3** *, is of length* **ln3** *, and for every existing noun* **n3** *, which follows comma* **c2** *, and for every existing noun* **n4** *, which precedes comma* **c2** *, has a start position* **sp4** *, is of length* **ln4** *, the only one coreference relation* **r** *, which is resolved in text* **t** *, is of “Pronominal” type, “Relative” subtype, “Backward” position and “Multiple” group, its referent starts at position* **sp1** *and has length* **ln1** *, fits only one lexeme* **p** *, refers to only one mention* **m1** *, which starts at position* **sp2** *, has length* **ln2** *, and fits noun* **n1** *, refers to only one mention* **m2** *, which starts at position* **sp3** *, has length* **ln3** *, and fits only one noun* **n2** *, and refers at least to one mention* **m3** *, which starts at position* **sp4** *, has length* **ln4,** *and fits noun* **n4** *, exists* (Rule 2).

  Rule 2: ∀t, s, p, l, c1, n1, sp1, ln1, sp2, ln2, j, n2, sp3, ln3.[Text(t) ⋀ Sentence(s) ⋀ consists_of(t, s) ⋀ Pronoun(p) ⋀ contains(s, p) ⋀ has_number(p, Plural) ⋀ has_type(p, Relative) ⋀ has_start_position(p, sp1) ⋀ has_length(p, ln1) ⋀ Comma(c1) ⋀ (follows(p, c1) ⋁ (Lexeme(l) ⋀ has_pos(l, Preposition) ⋀ follows(p, l) ⋀ follows(l, c1)) ⋀ Noun(n1) ⋀ follows(c1, n1) ⋀ has_start_position(n1, sp2) ⋀ has_length(n1, ln2) ⋀ Conjunction(j) ⋀ follows(n1, j) ⋀ Noun(n2) ⋀ follows(j, n2) ⋀ has_start_position(n2, sp3) ⋀ has_length(n2, ln3) ⋀ (∃n3, c2, n4, sp4, ln4.(Noun(n3) ⋀ Comma(c2) ⋀ Noun(n4) ⋀ follows(n3, c2) ⋀ follows(c2, n4) ⋀ has_start_position(n4, sp4) ⋀ has_length(n4, ln4)) ⟶ ∃!r ∃!m1 ∃!m2 ∃m3. [Coreference(r) ⋀ resolved_in(r, t) ⋀ has_type(r, Pronominal) ⋀ has_subtype(r, Relative) ⋀ has_position(r, Backward) ⋀ has_ group(r, Multiple) ⋀ has_start_position(r, sp1) ⋀ has_length(r, ln1) ⋀ fits(r, p) ⋀ Mention(m1) ⋀ refers_to(r, m1) ⋀ has_start_position(m1, sp2) ⋀ has_length(m1, ln2) ⋀ fits(m1, n1) ⋀ Mention(m2) ⋀ refers_to(r, m2) ⋀ has_start_position(m2, sp3) ⋀ has_length(m2, ln3) ⋀ fits(m2, n2) ⋀ Mention(m3) ⋀ refers_to(r, m3) ⋀ has_start_position(m3, sp4) ⋀ has_length(m3, ln4) ⋀ fits(m3, n4)]]

Though examples illustrating the certain case of coreference are given in Lithuanian and English only, rules for resolution of such coreferences could be applied for other languages as well. For example, Rule 1 could be successfully applied for coreference resolution in Polish or Russian languages. Let us take the same example of a sentence in Polish and Russian:

  [PL] Dzisiaj przychodził mężczyzna [noun], który [pronoun] skarżył się na ból pleców.
  [RU] Сегодня приходил мужчина [noun], который [pronoun] жаловался на боль в спине.

We can see that a structure of the sentence (number and order of lexemes) is similar, a pronoun goes after the comma and it refers to a noun, and compatibility of morphological features (gender, number) of noun and pronoun is retained. From the given example, we understand that the coreference relation between pronoun and noun exists and conditions for such existence are the same as specified in Rule 1.

#### 3.4.2. A2: General Purpose Pronoun Resolution

This algorithm focuses on the cases where pronouns refer to nouns (or noun phrases) that are recognized as named entities of “person” class by NER. The algorithm starts from the identification of not demonstrative pronoun. In a given example below, such a pronoun is in the second sentence—“Jis” (“He”)

- [LT] **Jonas Jonaitis** [person noun phrase] skambino į registratūrą. **Jis** [pronoun] skundėsi galvos skausmu.
- [EN] **Jonas Jonaitis** [person noun phrase] called a reception. **He** [pronoun] complained about headache.

If the pronoun is in the relative clause, the algorithm moves backwards analysing words going before the pronoun. In a given example, the pronoun is at the beginning of the sentence, so remaining parts of the sentence are not analysed, and the algorithm moves one sentence backwards.

The conditions for the existence of such reference formally could be defined as three alternatives. The first one describes conditions for reference existing in the same sentence **s1** before pronoun **p**:

*For each text's* **t** *sentence* **s1** *and pronoun* **p** *not of Demonstrative type that is contained in sentence* **s1** *and has gender* **g,** *number* **n** *, start position* **sp1** *and length of* **ln1** *, and named entity* **e1** *that is in the same sentence* **s1** *, is expressed by lexeme l, and has gender* **g** *, number* **n** *, start position* **sp2** *and is of length* **ln2** *, and is before pronoun* **p **(**sp2 ***is lower than ***sp1***), but closer to pronoun ***p ***than possible named entities ***e2 ***and ***e3 **(**sp2 ***higher than ***sp3 ***and ***sp4***), the only one coreference relation ***r***, which is resolved in text ***t***, is of “Pronominal” type, “Relative” subtype, “Backward” position and “Single” group between the pronoun ***p ***and the named entity ***e1***, its referent starts at position ***sp1 ***and has length ***ln1***, and which fits only one pronoun ***p ***and refers to only one mention ***m***, which starts at position ***sp2***, has length ***ln2***, and fits only one named entity ***e1***, exists* (Rule 3).

  Rule 3: ∀t, s1, p, l, e1, g, n, sp1, ln1, sp2, ln2.[Text(t) ⋀ Sentence(s1) ⋀ consists_of(t, s1) ⋀ Pronoun(p) ⋀ contains(s1, p) ⋀ ¬has_type(p, Demonstrative) ⋀ has_gender(p, g) ⋀ has_number(p, n) ⋀ has_start_position(p, sp1) ⋀ has_length(p, ln1) ⋀ Person_NE(e1) ⋀ includes(s1, e1) ⋀ Lexeme(l) ⋀ expressed_by(e1, l) ⋀ has_gender(e1, g) ⋀ has_number(e1, n) ⋀ has_start_position(e1, sp2) ⋀ has_length(e1, ln2) ⋀ sp2<sp1 ⋀ ¬(∃e2, e3, sp3, sp4. (e1≠e2 ⋀ e1≠e3 ⋀ e2≠e3 ⋀ Person_NE(e2) ⋀ includes(s1, e2) ⋀ has_gender(e2, g) ⋀ has_number(e2, n) ⋀ has_start_position(e2, sp3) ⋀ Person_NE(p3) ⋀ includes(s1, e3) ⋀ has_gender(e3, g) ⋀ has_number(e3, n) ⋀ has_start_position(e3, sp4) ⋀ sp2>sp3 ⋀ sp4>sp2)) ⟶ ∃!r ∃!m. [Coreference(r) ⋀ resolved_in(r, t) ⋀ has_type (r, Pronominal) ⋀ has_subtype (r, General) ⋀ has_position(r, Backward) ⋀ has_group(r, Single) ⋀ has_start_position(r, sp1) ⋀ has_length(r, ln1) ⋀ fits(r, p) ⋀ Mention(m) ⋀ refers_to(r, t) ⋀ has_start_position(m, sp2) ⋀ has_length(m, ln2) ⋀ fits(m, e1) ⋀ fits(m, l)]]

The second alternative describes a case when a pronoun **p** refers to the named entity in the previous sentence **s2**:

*For each text's* **t** *sentence* **s1, s2** *, where* **s1** *follows* **s2** *, and pronoun* **p** *not of Demonstrative type that is contained in sentence* **s1** *and has gender* **g,** *number* **n** *, start position* **sp1** *and length of* **ln1** *, and named entity* **e1** *that is contained in sentence* **s2** *, is expressed by lexeme l, and has gender* **g** *, number* **n**, *start position ***sp2 ***and is of length ***ln2***, and is closer to pronoun ***p ***than possible named entities ***e2 ***and ***e3 **(**sp2 ***higher than ***sp3 ***and ***sp4***), the only one coreference relation ***r***, which is resolved in text ***t***, is of “Pronominal” type, “Relative” subtype, “Backward” position and “Single” group between the pronoun ***p ***and the named entity ***e1***, its referent starts at position ***sp1 ***and has length ***ln1***, and which fits only one pronoun ***p ***and refers to only one mention ***m***, which starts at position ***sp2***, has length ***ln2***, and fits only one named entity ***e1***, exists* (Rule 4).

  Rule 4: ∀t, s1, s2, p, l, e1, g, n, sp1, ln1, sp2, ln2.[Text(t) ⋀ Sentence(s1) ⋀ Sentence(s2) ⋀ consists_of(t, s1) ⋀ consists_of(t, s2) ⋀ follows (s1, s2) ⋀ Pronoun(p) ⋀ contains(s1, p) ⋀ ¬has_type(p, Demonstrative) ⋀ has_gender(p, g) ⋀ has_number(p, n) ⋀ has_start_position(p, sp1) ⋀ has_length(p, ln1) ⋀ Person_NE(e1) ⋀ includes(s2, e1) ⋀ Lexeme(l) ⋀ expressed_by(e1, l) ⋀ has_gender(e1, g) ⋀ has_number(e1, n) ⋀ has_start_position(e1, sp2) ⋀ has_length(e1, ln2) ⋀ ¬(∃e2, e3, sp3, sp4. (e1≠e2 ⋀ e1≠e3 ⋀ e2≠e3 ⋀ Person_NE(e2) ⋀ includes(s2, e2) ⋀ has_gender(e2, g) ⋀ has_number(e2, n) ⋀ has_start_position(e2, sp3) ⋀ Person_NE(p3) ⋀ includes(s2, e3) ⋀ has_gender(e3, g) ⋀ has_number(e3, n) ⋀ has_start_position(e3, sp4) ⋀ sp2>sp3 ⋀ sp4>sp2)) ⟶ ∃!r ∃!m. [Coreference(r) ⋀ resolved_in(r, t) ⋀ has_type (r, Pronominal) ⋀ has_subtype (r, General) ⋀ has_position(r, Backward) ⋀ has_group(r, Single) ⋀ has_start_position(r, sp1) ⋀ has_length(r, ln1) ⋀ fits(r, p) ⋀ Mention(m) ⋀ refers_to(r, t) ⋀ has_start_position(m, sp2) ⋀ has_length(m, ln2) ⋀ fits(m, e1) ⋀ fits(m, l)]]

The third alternative describes a case when a pronoun **p** in the sentence **s1** refers to the named entity in the sentence **s3**, preceding sentences **s2** and **s1**:

*For each text's* **t** *sentence* **s1**, **s2**, **s3***, where ***s1 ***follows ***s2 ***and ***s2 ***follows ***s3***, and pronoun ***p ***not of Demonstrative type that is contained in sentence ***s1 ***and has gender ***g**, *number ***n***, start position ***sp1 ***and length of ***ln1***, and named entity ***e1 ***that is contained in sentence ***s3***, is expressed by lexeme l, and has gender ***g***, number ***n**, *start position ***sp2 ***and is of length ***ln2***, and is closer to pronoun ***p ***than possible named entities ***e2 ***and ***e3 **(**sp2 ***higher than ***sp3 ***and ***sp4***), the only one coreference relation ***r***, which is resolved in text ***t***, is of “Pronominal” type, “Relative” subtype, “Backward” position and “Single” group between the pronoun ***p ***and the named entity ***e1***, its referent starts at position ***sp1 ***and has length ***ln1***, and which fits only one pronoun ***p ***and refers to only one mention ***m***, which starts at position ***sp2***, has length ***ln2***, and fits only one named entity ***e1***, exists* (Rule 5).

  Rule 5: ∀t, s1, s2, s3, p, l, e1, g, n, sp1, ln1, sp2, ln2.[Text(t) ⋀ Sentence(s1) ⋀ Sentence(s2) ⋀ Sentence(s3) ⋀ consists_of(t, s1) ⋀ consists_of(t, s2) ⋀ consists_of(t, s3) ⋀ follows (s1, s2) ⋀ follows (s2, s3) ⋀ Pronoun(p) ⋀ contains(s1, p) ⋀ ¬has_type(p, Demonstrative) ⋀ has_gender(p, g) ⋀ has_number(p, n) ⋀ has_start_position(p, sp1) ⋀ has_length(p, ln1) ⋀ Person_NE(e1) ⋀ Lexeme(l) ⋀ expressed_by(e1, l) ⋀ includes(s3, e1) ⋀ has_gender(e1, g) ⋀ has_number(e1, n) ⋀ has_start_position(e1, sp2) ⋀ has_length(e1, ln2) ⋀ ¬(∃e2, e3, sp3, sp4. (e1≠e2 ⋀ e1≠e3 ⋀ e2≠e3 ⋀ Person_NE(e2) ⋀ includes(s3, e2) ⋀ has_gender(e2, g) ⋀ has_number(e2, n) ⋀ has_start_position(e2, sp3) ⋀ Person_NE(e3) ⋀ includes(s3, e3) ⋀ has_gender(e3, g) ⋀ has_number(e3, n) ⋀ has_start_position(e3, sp4) ⋀ sp2>sp3 ⋀ sp4>sp2)) ⟶ ∃!r ∃!m. [Coreference(r) ⋀ resolved_in(r, t) ⋀ has_type (r, Pronominal) ⋀ has_subtype (r, General) ⋀ has_position(r, Backward) ⋀ has_group(r, Single) ⋀ has_start_position(r, sp1) ⋀ has_length(r, ln1) ⋀ fits(r, p) ⋀ Mention(m) ⋀ refers_to(r, t) ⋀ has_start_position(m, sp2) ⋀ has_length(m, ln2) ⋀ fits(m, e1) ⋀ fits(m, l)]]

Another example presents a case when a coreferent of the pronoun “man” (in English, “for me”) is in the following sentence:

- [LT] Pastebėtina, kad ligoginėse apsilankė 10 mln. pacientų, nepaisant to, kad 2016 m. jų buvo 4% mažiau (apsilankė beveik 9,5 mln.). Tai labiausiai lėmė skaitmeninių paslaugų padidinimas: „Kiek **man** [pronoun] teko analizuoti, padidinus skaitmenines paslaugas tik nedidelė dalis **Lietuvos** [location noun] ligoninių sumažino etatų ar atleido darbuotojus, o tai lėmė nemažą papildomą indėlį į paslaugos kokybę” teigė J. **Jonaitis** [person noun phrase].
- [EN] It is noteworthy that the total hospital patient count has reached 10 million, even though in 2016 the number was less than 4% (around 9.5 million patients). This was influenced by the digitization of e-health services. “As far as **I** [pronoun] had analysed, only a small part of **Lithuanian** [location noun] hospitals have reduced their posts or dismissed employees, but digitization of services has led to a significant additional contribution to the quality of service,” said J. **Jonaitis** [person noun phrase].

If the algorithm does not find any named entities moving backwards, it moves back to pronoun and proceeds forward. The algorithm continues moving forward until it locates “J. Jonaitis” entity, which is recognized as a person. Since the gender of the pronoun “man” is ambiguous (it can refer to both female and male persons), only their grammatical numbers are compared. Both are singular; therefore, the algorithm picks “J. Jonaitis” as a postcedent of the corefering object “man.” Conditions for the existence of such reference formally could be defined as two alternatives. The first one describes the conditions for reference existing in the same sentence **s1** after pronoun was mentioned:

*For each text's* **t** *sentence* **s1** *and pronoun* **p** *not of Demonstrative type that is contained in sentence* **s1** *and has gender* **g,** *number* **n** *, start position* **sp1** *and length of* **ln1** *, and named entity* **e1** *that is in the same sentence* **s1** *, is expressed by lexeme l, and has gender* **g** *, number* **n** *, start position* **sp2** *and is of length* **ln2** *, and is after pronoun* **p **(**sp2 ***is higher than ***sp1***), but closer to pronoun ***p ***than possible named entities ***e2 ***and ***e3 **(**sp2 ***higher than ***sp3 ***and ***sp4***), the only one coreference relation ***r***, which is resolved in text ***t***, is of “Pronominal” type, “Relative” subtype, “Backward” position and “Single” group between the pronoun ***p ***and the named entity ***e1***, its referent starts at position ***sp1 ***and has length ***ln1***, and which fits only one pronoun ***p ***and refers to only one mention ***m***, which starts at position ***sp2***, has length ***ln2***, and fits only one named entity ***e1***, exists* (Rule 6).

  Rule 6: ∀t, s1, p, l, e1, g, n, sp1, ln1, sp2, ln2.[Text(t) ⋀ Sentence(s1) ⋀ consists_of(t, s1) ⋀ Pronoun(p) ⋀ contains(s1, p) ⋀ ¬has_type(p, Demonstrative) ⋀ has_gender(p, g) ⋀ has_number(p, n) ⋀ has_start_position(p, sp1) ⋀ has_length(p, ln1) ⋀ Person_NE(e1) ⋀ includes(s1, e1) ⋀ Lexeme(l) ⋀ expressed_by(e1, l) ⋀ has_gender(e1, g) ⋀ has_number(e1, n) ⋀ has_start_position(e1, sp2) ⋀ has_length(e1, ln2) ⋀ sp1<sp2 ⋀ ¬(∃e2, e3, sp3, sp4. (e1≠e2 ⋀ e1≠e3 ⋀ e2≠e3 ⋀ Person_NE(e2) ⋀ includes(s1, e2) ⋀ has_start_position(e2, sp3) ⋀ Person_NE(e3) ⋀ includes(s1, e3) ⋀ has_start_position(e3, sp4) ⋀ sp2<sp3 ⋀ sp4<sp2)) ⟶ ∃!r ∃!m. [Coreference(r) ⋀ resolved_in(r, t) ⋀ has_type (r, Pronominal) ⋀ has_subtype (r, General) ⋀ has_position(r, Forward) ⋀ has_group(r, Single) ⋀ has_start_position(r, sp1) ⋀ has_length(r, ln1) ⋀ fits(r, p) ⋀ Mention(m) ⋀ refers_to(r, t) ⋀ has_start_position(m, sp2) ⋀ has_length(m, ln2) ⋀ fits(m, e1) ⋀ fits(m, l)]]

The second alternative describes the case when the pronoun **p** refers to the named entity in the following sentence **s4**:

*For each text's* **t** *sentence* **s1, s4,** *where* **s4** *follows* **s1** *, and pronoun* **p** *not of Demonstrative type that is contained in sentence* **s1** *and has gender* **g**, *number ***n***, start position ***sp1 ***and length of ***ln1***, and named entity ***e1 ***that is contained in sentence ***s2***, is expressed by lexeme l, and has gender ***g***, number ***n***, start position ***sp2 ***and is of length ***ln2***, and is closer to pronoun ***p ***than possible named entities ***e2 ***and ***e3 ***( ***sp2 ***higher than ***sp3 ***and ***sp4 ***), the only coreference relation ***r***, which is resolved in text ***t***, is of “Pronominal” type, “Relative” subtype, “Backward” position and “Single” group between the pronoun ****p ****and the named entity ***e1***, its referent starts at position ***sp1 ***and has length ***ln1***, and which fits only one pronoun ***p ***and refers to only one mention ***m***, which starts at position ***sp2***, has length ***ln2***, and fits only one named entity ***e1***, exists* (Rule 7).

  Rule 7: ∀t, s1, s2, p, l, e1, g, n, sp1, ln1, sp2, ln2.[Text(t) ⋀ Sentence(s1) ⋀ Sentence(s2) ⋀ consists_of(t, s1) ⋀ consists_of(t, s2) ⋀ follows (s2, s1) ⋀ Pronoun(p) ⋀ contains(s1, p) ⋀ ¬has_type(p, Demonstrative) ⋀ has_gender(p, g) ⋀ has_number(p, n) ⋀ has_start_position(p, sp1) ⋀ has_length(p, ln1) ⋀ Person_NE(e1) ⋀ includes(s2, e1) ⋀ Lexeme(l) ⋀ expressed_by(e1, l) ⋀ has_gender(e1, g) ⋀ has_number(e1, n) ⋀ has_start_position(e1, sp2) ⋀ has_length(e1, ln2) ⋀ ¬(∃e2, e3, sp3, sp4. (e1≠e2 ⋀ e1≠e3 ⋀ e2≠e3 ⋀ Person_NE(e2) ⋀ includes(s2, e2) ⋀ has_start_position(e2, sp3) ⋀ Person_NE(e3) ⋀ includes(s2, e3) ⋀ has_start_position(e3, sp4) ⋀ sp2<sp3 ⋀ sp4<sp2)) ⟶ ∃!r ∃!m. [Coreference(r) ⋀ resolved_in(r, t) ⋀ has_type (r, Pronominal) ⋀ has_subtype (r, General) ⋀ has_position(r, Forward) ⋀ has_group(r, Single) ⋀ has_start_position(r, sp1) ⋀ has_length(r, ln1) ⋀ fits(r, p) ⋀ Mention(m) ⋀ refers_to(r, t) ⋀ has_start_position(m, sp2) ⋀ has_length(m, ln2) ⋀ fits(m, e1) ⋀ fits(m, l)]]

The algorithm ignores demonstrative pronouns because they are often used to refer to entities that are not present in the written text. Such pronouns do not carry any additional semantic information and do not refer to any noun phrase. They are used mostly for syntactic reasons and due to that are usually ignored in coreference resolution.

#### 3.4.3. A3: PRA Resolution

This algorithm is based on exact (or partial) string matches and several rules for acronyms. Once a first named entity that can be matched with an initial named entity is found, then the algorithm stops to keep annotations simple: B ⟶ A, C ⟶ B and D ⟶ C. This allows the formation of the coreference chains linking all mentions of the same entity in a text that can be later reused for semantic analysis, for example,

- [LT] **Tomaitis** [named entity] pateko į avariją. Po pietų **Tomaitį** [named entity] išvežė į operacinę.
- [EN] **Tomaitis** [named entity] got into a car accident. In the afternoon, **Tomaitis** [named entity] has been taken to a surgery room.

In this example, two mentions of the same entity are made: “Tomaitis” and “Tomaitį.” They are of different cases, but their lemmas are identical. A condition for the existence of such reference formally is defined as follows:

*For each text's* **t** *sentence* **s1** *that includes named entity* **e1** *, that has start position* **sp1** *and is of length* **ln1** *, which is expressed by lexeme* **l1** *that has lemma* **l** *and for each same text's* **t** *sentence* **s2** *that includes named entity* **e2** *, that has a start position* **sp1** *and is of length* **ln1** *, which is expressed by lexeme* **l2** *that has lemma* **l** *, the only one coreference relation* **r** *, which is resolved in text* **t** *, is of “Nominal” type, “Repetition” subtype, “Irrelevant” position and “Single” group between the noun* **n1** *and the noun* **n2** *, its referent starts at position* **sp1** *and has length* **ln1** *, and which fits only one noun* **n1** *and refers to only one mention* **m** *, which starts at position* **sp2** *, has length* **ln2** *, and fits only one noun* **n2** *, exists* (Rule 8).

  Rule 8: ∀t, s1, s2, e1, e2, sp1, ln1, sp2, ln2.[Text(t) ⋀ Sentence(s1) ⋀ Sentence(s2) ⋀ consists_of(t, s1) ⋀ consists_of(t, s2) ⋀ Named_Entity(e1) ⋀ includes(s1, e1) ⋀ has_start_position(e1, sp1) ⋀ has_length(e1, ln1) ⋀ Named_Entity(e2) ⋀ includes(s2, e2) ⋀ has_start_position(e2, sp2) ⋀ has_length(e2, ln2) ⋀ e1≠e2 ⋀ (∃l1 ∃l2 ∃l.(Lexeme(l1) ⋀ Lexeme(l2) ⋀ expressed_by(e1, l1) ⋀ expressed_by(e2, l2) ⋀ has_lemma(l1, l) ⋀ has_lemma(l2, l)) ⟶ ∃!r ∃!m. [Coreference(r) ⋀ resolved_in(r, t) ⋀ has_type (r, Nominal) ⋀ has_subtype (r, Repetition) ⋀ has_position(r, Irrelevant) ⋀ has_group(r, Single) ⋀ has_start_position(r, sp1) ⋀ has_length(r, ln1) ⋀ fits(r, l1) ⋀ fits(r, e1) ⋀ Mention(m) ⋀ refers_to(r, t) ⋀ has_start_position(m, sp2) ⋀ has_length(m, ln2) ⋀ fits(m, l2) ⋀ fits(m, e2)]]

Acronym rules vary depending on the type of named entity (currently persons, locations, and organizations are covered).

#### 3.4.4. A4: HHS Resolution

This algorithm is based on profession classification. It attempts to resolve the use of synonyms and hypernyms/hyponyms.

- [LT] **Gydytojai** [noun referring to profession] skundžiasi dideliu darbo krūviu. **Chirurgų** [noun referring to profession] darbo krūvis pats didžiausias.
- [EN] **Doctors** [noun referring to profession] complain about heavy workload. **Surgeons'** [noun referring to profession] workload is the greatest.

The algorithm determines that “Doctor” in professions classification is a hyponym of “Surgeon,” they also agree in gender and number; therefore, the algorithm adds their pair to annotations. Conditions for the existence of such reference formally are defined as follows:

*For each text's* **t** *sentence* **s1** *that has profession* **p1** *, which is either broader or narrower than profession* **p2** *, name* **v1** *expressing noun* **n1** *, which has gender* **g** *, number* **m** *, start position* **sp1** *and is of length* **ln1** *, and for each same text's* **t** *sentence* **s2** *that has profession* **p2** *, which is either broader or narrower than profession* **p1** *, name* **v2** *expressing noun* **n2** *, which has gender* **g** *, number* **m** *, start position* **sp2** *and is of length* **ln2** *, the only one coreference relation* **r** *, which is resolved in text* **t** *, is of “Nominal” type, “Hypernym_hyponym” subtype, “Irrelevant” position and “Single” group between the noun* **n1** *and the noun* **n2** *, its referent starts at position* **sp1** *and has length* **ln1** *, and which fits only one noun* **n1** *and refers to only one mention* **m** *, which starts at position* **sp2** *, has length* **ln2** *, and fits only one noun* **n2** *, exists* (Rule 9).

  Rule 9: ∀t, s1, s2, n1, n2, sp1, ln1, sp2, ln2, v1, v2, p1, p2.[Text(t) ⋀ Sentence(s1) ⋀ Sentence(s2) ⋀ consists_of(t, s1) ⋀ consists_of(t, s2) ⋀ Noun(n1) ⋀ contains(s, n1) ⋀ has_start_position(n1, sp1) ⋀ has_length(n1, ln1) ⋀ Noun(n2) ⋀ contains(s2, n2) ⋀ has_start_position(n2, sp2) ⋀ has_length(n2, ln2) ⋀ n1≠n2 ⋀ Profession(p1) ⋀ Profession(p2) ⋀ p1≠p2 ⋀ Profession_Name(v1) ⋀ Profession_Name(v2) ⋀ express(n1, v1) ⋀ express(n2, v2) ⋀ describes(v1, p1) ⋀ describes(v2, p2) ⋀ (broadens(p2, p1) ⋁ broadens(p1, p2)) ⋀ has_gender(n1, g) ⋀ has_gender(n2, g) ⋀ has_number(n1, n) ⋀ has_number(n2, n) ⟶ ∃!r ∃!m. [Coreference(r) ⋀ resolved_in(r, t) ⋀ has_type (r, Nominal) ⋀ has_subtype (r, Hypernym_Hyponym) ⋀ has_position(r, Irrelevant) ⋀ has_group(r, Single) ⋀ has_start_position(r, sp1) ⋀ has_length(r, ln1) ⋀ fits(r, n1) ⋀ Mention(m) ⋀ refers_to(r, t) ⋀ has_start_position(m, sp2) ⋀ has_length(m, ln2) ⋀ fits(m, n2)]]

An example of synonym is given as follows:

- [LT] J. Jonaitis buvo operacijos **vadovu** [noun referring to profession]. Deja, **vyr. chirurgo** [noun referring to profession] vykdoma operacija buvo nesėkminga.
- [EN] From now J. Jonaitis was the **head** surgeon [noun referring to profession] of operation. Unfortunately, the last operation of **chief surgeon** [noun referring to profession] was unsuccessful.

Both “head surgeon” and “chief surgeon” are synonymous; therefore, the condition for the existence of such reference formally could be defined as follows:

*For each text's* **t** *sentence* **s1** *that has a profession's* **p** *name* **v1** *, which is expressed by noun* **n1** *that has gender* **g** *, number* **m** *, start position* **sp1** *and is of length* **ln1** *, and for each same text's* **t** *sentence* **s2** *that has same profession's* **p** *name* **v2** *expressed by noun* **n2** *that has gender* **g** *, number* **m** *, start position* **sp2** *and is of length* **ln2** *, the only one coreference relation* **r** *, which is resolved in text* **t** *, is of “Nominal” type, “Synonym” subtype, “Irrelevant” position and “Single” group between the noun* **n1** *and the noun* **n2** *, its referent starts at position* **sp1** *and has length* **ln1** *, and which fits only one noun* **n1** *and refers to only one mention* **m** *, which starts at position* **sp2** *, has length* **ln2** *, and fits only one noun* **n2** *, exists* (Rule 10).

  Rule 10: ∀t, s1, s2, n1, n2, sp1, ln1, sp2, ln2, v1, v2, p, g, n.[Text(t) ⋀ Sentence(s1) ⋀ Sentence(s2) ⋀ consists_of(t, s1) ⋀ consists_of(t, s2) ⋀ Noun(n1) ⋀ contains(s1, n1) ⋀ has_start_position(n1, sp1) ⋀ has_length(n1, ln1) ⋀ Noun(n2) ⋀ contains(s2, n2) ⋀ has_start_position(n2, sp2) ⋀ has_length(n2, ln2) ⋀ n1≠n2 ⋀ Profession_name(v1) ⋀ Profession_name(v2) ⋀ Profession(p) ⋀ express(n1, v1) ⋀ express(n2, v2) ⋀ describes(v1, p) ⋀ describes(v2, p) ⋀ has_gender(n1, g) ⋀ has_gender(n2, g) ⋀ has_number(n1, n) ⋀ has_number(n2, n) ⋀ n1≠n2 ⟶ ∃!r ∃!m. [Coreference(r) ⋀ resolved_in(r, t) ⋀ has_type (r, Nominal) ⋀ has_subtype(r, Synonym) ⋀ has_position(r, Irrelevant) ⋀ has_group(r, Single) ⋀ has_start_position(r, sp1) ⋀ has_length(r, ln1) ⋀ fits(r, n1) ⋀ Mention(m) ⋀ refers_to(r, t) ⋀ has_start_position(m, sp2) ⋀ has_length(m, ln2) ⋀ fits(m, n2)]]

#### 3.4.5. A5: Feature Resolution

This algorithm at the time attempts to resolve only those cases when a person is being referred to by his public post (feature) that he holds, other types of features are not currently resolved, for example,

- [LT] Ką rekomenduoja S. Suskelis [person noun phrase]? Aptarkime kardiologo [noun referring to held position] siūlomą gydymo planą.
- [EN] What does S. Suskelis [person noun phrase] recommend? Let's discuss treatment plan proposed by the cardiologist [noun referring to held position]. Here a noun “cardiologist” is selected, the algorithm moves backwards till it reaches “S. Suskelis” and checks the knowledge base if at the time of the publication of the medical record he has held the position of the cardiologist.. Since “he holds it” the algorithm checks if “S. Suskelis” and “cardiologist” agree in gender and number. They agree, and their pair is added to annotation as a feature reference. A condition for the existence of such reference formally is defined as follows:

*For each text's* **t** *sentence* **s1** *that has known person* **k** *, who during publication date* **d** *had certain position* **h** *(publication date* **d** *is same or later than position* **h** *start date* **fd** *and same or earlier than position* **h** *end date* **td** *), mention as named entity* **e** *, that has a start position* **sp1** *and is of length* **ln1** *, and for each same text's* **t** *sentence* **s2** *mentioned noun* **n** *, that has a start position* **sp2** *and is length* **ln2** *, which is mentioned after named entity* ***e*** *(noun* **n** *has a higher start position* **sp2** *than named entity's* **sp1** *), whose lemma* **l** *matches with position's* **h** *lemma* **l** *, number is Singular and gender g matches known person's* **k** *gender* **g** *, the only one coreference relation* **r** *, which is resolved in text* **t** *, is of “Nominal” type, “Feature” subtype, “Backward” position and “Single” group between the noun* **n** *and the named entity* **e** *, its referent starts at position* **sp2** *and has length* **ln2** *, and which fits only one noun* **n** *and refers to only one mention* **m** *, which starts at position* **sp1** *, has length* **ln1** *, and fits only one named entity* **e** *, exists* (Rule 11).

  Rule 11: ∀t, s1, s2, n, k, h, l, d, fd, td, g, sp1, sp2, ln1, ln2.[Text(t) ⋀ Sentence(s1) ⋀ Sentence(s2) ⋀ consists_of(t, s1) ⋀ consists_of(t, s2) ⋀ Person_NE(e) ⋀ includes(s1, e) ⋀ Noun(n) ⋀ contains(s2, n) ⋀ Known_Person(k) ⋀ mentioned_as(k, e) ⋀ Position_held(h) ⋀ holds(k, h) ⋀ has_lemma(h, l) ⋀ has_lemma(n, l) ⋀ has_publication_date(t, d) ⋀ has_from_date(h, fd) ⋀ has_to_date(h, td) ⋀ fd≤d ⋀ td≥d ⋀ has_gender(k, g) ⋀ has_gender(n, g) ⋀ has_number(n, Singular) ⋀ has_start_position(e, sp1) ⋀ has_start_position(n, sp2) ⋀ sp1<sp2 ⋀ has_length(e, ln1) ⋀ has_length(n, ln2) ⟶ ∃!r ∃!m. [Coreference(r) ⋀ resolved_in(r, t) ⋀ has_type (r, Nominal) ⋀ has_subtype (r, Feature) ⋀ has_position(r, Backward) ⋀ has_ group(r, Single) ⋀ has_start_position(r, sp2) ⋀ has_length(r, ln2) ⋀ fits(r, e) ⋀ Mention(m) ⋀ refers_to(r, t) ⋀ has_start_position(m, sp1) ⋀ has_length(m, ln1) ⋀ fits(m, n)]]

In this case, it is also relevant to track if coreference is pointing backward or forward. We can rewrite the same example and switch a known person with his positions:

- [LT] Koks yra mano **šeimos gydytojas** [noun referring to held position]? T. **Tomaitis** [person noun phrase] yra labai patyręs jūsų šeimos gydytojas.
- [EN] Who is my **family doctor** [noun referring to held position]? T. **Tomaitis** [person noun phrase] is very experienced doctor assigned to you.

As a result, **sp1** is higher than **sp2** and coreference has different position constant values:

*For each text's* **t** *sentence* **s1** *that has known person* **k** *, who during publication date* **d** *had certain position* **h** *(publication date* **d** *is same or later than position* **h** *start date* **fd** *and same or earlier than position* **h** *end date* **td** *), mention as named entity* **e** *, that has a start position* **sp1** *and is of length* **ln1** *, and for each same text's* **t** *sentence* **s2** *mentioned noun* **n** *, that has a start position* **sp2** *and is length* **ln2** *, which is mentioned after named entity* **e** *(noun* **n** *has a lower start position* **sp2** *than named entity's* **sp1** *), whose lemma* **l** *matches with position's* **h** *lemma* **l** *, number is Singular and gender g matches known person's* **k** *gender* **g** *, the only one coreference relation* **r** *, which is resolved in text* **t** *, is of “Nominal” type, “Feature” subtype, “Forward” position and “Single” group between the noun* **n** *and the named entity* **e** *, its referent starts at position* **sp2** *and has length* **ln2** *, and which fits only one noun* **n** *and refers to only one mention* **m** *, which starts at position* **sp1** *, has length* **ln1** *, and fits only one named entity* **e** *, exists* (Rule 12).

  Rule 12: ∀t, s1, s2, n, k, h, l, d, fd, td, g, sp1, sp2, ln1, ln2.[Text(t) ⋀ Sentence(s1) ⋀ Sentence(s2) ⋀ consists_of(t, s1) ⋀ consists_of(t, s2) ⋀ Person_NE(e) ⋀ includes(s1, e) ⋀ Noun(n) ⋀ contains(s2, n) ⋀ Known_Person(k) ⋀ mentioned_as(k, e) ⋀ Position_held(h) ⋀ holds(k, h) ⋀ has_lemma(h, l) ⋀ has_lemma(n, l) ⋀ has_publication_date(t, d) ⋀ has_from_date(h, fd) ⋀ has_to_date(h, td) ⋀ fd≤d ⋀ td≥d ⋀ has_gender(k, g) ⋀ has_gender(n, g) ⋀ has_number(n, Singular) ⋀ has_start_position(e, sp1) ⋀ has_start_position(n, sp2) ⋀ sp1>sp2 ⋀ has_length(e, ln1) ⋀ has_length(n, ln2) ⟶ ∃!r ∃!m. [Coreference(r) ⋀ resolved_in(r, t) ⋀ has_type (r, Nominal) ⋀ has_subtype (r, Feature) ⋀ has_position(r, Forward) ⋀ has_ group(r, Single) ⋀ has_start_position(r, sp2) ⋀ has_length(r, ln2) ⋀ fits(r, e) ⋀ Mention(m) ⋀ refers_to(r, t) ⋀ has_start_position(m, sp1) ⋀ has_length(m, ln1) ⋀ fits(m, n)]]

## 4. Results and Evaluation

The coreference resolution algorithms and rules presented in Section 3 were implemented as a separate component and integrated into the semantic search framework NLP pipeline (Figure 1) because it requires lexical, morphological, and NE annotations of the text should be analysed. Solutions for other languages should not follow the same NLP pipeline architecture. But a supply of coreference resolution component with lexical, morphological, and NE information of the text must be ensured.

Coreference resolution for Lithuanian was implemented using Java programming language and JSON data format for annotation storage. But the proposed approach is not technology dependent, and for other languages, it can be implemented on any other platform.

The evaluation was performed by analysing 100 articles that have been preannotated and are available in our Lithuanian Language Coreference Corpus [55], in addition to the transcribed records of medical reception, which we cannot disclose due to the privacy requirements.

For evaluation, we used precision, recall, and *F*1 metrics. Recall *R* is the ratio of correctly resolved anaphoric expressions *C* to the total number of anaphoric expressions *T*. Precision *P* is the ratio of correctly resolved anaphoric expressions *C* to the number of resolved anaphoric expressions *F*. *F*1 is a harmonic mean of *P* and *R*:

(1) $$\begin{array}{c} R=\frac{C}{T},\\ P=\frac{C}{F},\\ F1=2\;*\;\frac{R\;*\;P}{\mathrm{R+P}}. \end{array}$$

Five experiments were performed with different combinations of coreferencing algorithms presented in Section 3. The results of the experiments are presented in Table 3.

Note the following threats to validity of our results:

- The database of public persons must be constantly updated as new information becomes available. Otherwise, recall will get noticeably lower when annotating newer texts.
- In the case where plural pronouns and nouns are used, they are difficult to be identified because of many variations possible that often ignore grammatical compatibility rules.

Linking the named entity to the position held taking into account the date of the publication of the text is limited considering that the text might be published today but written about things that happened in the past. There are no tools, which can identify the timeframe of a certain part of the text.

## 5. Conclusion

Medical entity recognition and coreferencing are difficult tasks in Lithuanian natural language processing (NLP). We proposed the coreference resolution approach for the Lithuanian language. The coreference resolution algorithm depends on morphological and named entity recognition (NER) annotations and preexisting databases. Due to the proposed approach being detached from specific implementation and rules being formalized, it would not be difficult to adapt it for grammatically similar languages. Our novelty is the ability to process coreferences with minimal linguistic resources, which are very important to consider in linguistic applications for under-resourced and endangered languages. While the proposed method provides encouraging results, when analysing transcribed medical records and other corpora, and they are comparable to the results achieved by other authors applying different resolution approaches on other languages, it has certain limitations: it is domain specific and is able to resolve only a subset of coreference types, while the relatively small dataset was used for experiments. Nevertheless, we hope that our method can contribute to the sustainable development of the NLP-powered online healthcare services in Lithuania.

## Figures and Tables

**Table 1:** Comparison of coreference resolution approaches.

Method	Foundation	Precision	Recall	*F*1
Hobbs [32]	Syntactic tree with labeled nodes, syntactic rules, selection constraint rules	0.81–0.91	na	na
BFP [33]	Centering theory	0.49–0.90	na	na
Left-right centering [34]	Modified centering theory	0.72–0.81	na	na
Mitkov [35]	POS tagger, antecedent indicators	0.897	na	na
RAP [36]	Salience factors	0.85–0.89	na	na
Xrenner [37]	Syntactic and semantic rules	0.51–0.55	0.49–0.57	0.49–0.56
Probabilistic [38]	Bayesian rule	0.82–0.84	na	na
MARS [39]	Genetic algorithms	0.53–0.84	na	na
Soon et al. [40]	Machine learning (decision tree C5)	0.65–0.69	0.53–0.56	0.62
ILP [41]	Machine learning (logistic classifier)	0.78–0.89	0.47–0.58	0.61–0.68
Wiseman et al. [42]	Deep learning	0.77	0.70	0.73
Lee et al. [43]	Deep learning	0.81	0.73	0.77
Žitnik et al. [44]	Conditional random fields	0.68–0.94	0.30–0.87	0.41–0.87

**Table 2:** Comparison of coreference resolution methods for Balto-Slavic languages.

Method	Foundation	Precision	Recall	*F*1
LVCoref [45]	Rule based, Hobbs' algorithm	0.69–0.88	0.66–0.80	0.68–0.84
Ruler [46]	Rule based	0.59–0.65	0.50–0.75	0.55–0.69
BARTEK [47]	Machine learning	0.58	0.65	0.61
Mixed [48]	Deep learning, sieve based	0.70	0.68	0.69
RU-sys1 [49]	Rule based, ontology	0.82	0.70	0.76
RU-sys2 [49]	Rule based	0.71	0.58	0.64
RU-sys3 [49]	Rule based	0.63	0.50	0.55
RU-sys4 [49]	Statistical, ontology	0.54	0.51	0.53
RU-sys5 [49]	Machine learning, semantics	0.58	0.42	0.49
RU-sys6 [49]	Decision tree	0.36	0.15	0.21
Khadzhiiskaia and Sysoev [50]	Machine learning	0.84	0.77	0.80
Kučová and Žabokrtský [51]	Rule-based filters	0.60	na	na
CZ classifier [52]	Classifier-based machine learning	0.70–0.76	0.70–0.76	0.70–0.76
CZ ranker [52]	Ranker-based machine learning	0.79	0.79	0.79
Treex CR (Czech, English) [53]	Machine learning	na	na	0.61–0.68
Treex CR (Russian, German) [54]	Machine learning, projection	0.50–0.64	0.15–0.24	0.25–0.34

**Table 3:** Experiment results.

Algorithm	*T* (actual number of solvable expressions)	*F* (number of solvable expressions resolved by the algorithm)	*C* (number of solvable expressions correctly resolved by the algorithm)	*R* (recall)	*P* (precision)	*F*1
General purpose (GP)	648	248	202	0.311	0.814	0.451
GP + specific usage rules (SUR)	648	277	223	0.344	0.805	0.482
GP + SUR + PRA	648	330	267	0.412	0.809	0.546
GP + SUR + PRA + HHS	648	343	274	0.422	0.799	0.553
GP + SUR + PRA + HHS + feature	648	371	289	0.446	0.779	0.567

## Data Availability

The dataset used in this research is available upon request.
